# Supplementary material for: The genome sequence of the Woodland Grayling, Hipparchia fagi (Scopoli, 1763) (Lepidoptera: Nymphalidae)
Source: Wellcome Open Res. 2026 Feb 16;11:126. [Version 1] doi: 10.12688/wellcomeopenres.25873.1 (PMC13133624; doi:10.12688/wellcomeopenres.25873.1)
Supplement: Peer review history

# Reviewer report: Reviewer response for version 1

**DOI:** 10.21956/wellcomeopenres.28477.r151655

**Published:** 2026-04-30

**Authors:** Jose Martinez

**Affiliations:** 1 University of Florida, Gainesville, Florida, USA

*Hipparchia fagi* (Scopoli, 1763), commonly known as the Woodland Grayling, has been the subject of a recent genome assembly by Vila et al. This assembly shows a high degree of completeness, reflected in a BUSCO score of 98.6%, and is structured into 29 chromosomal pseudomolecules, including a fully assembled Z sex chromosome. The use of well-established and rigorous methodologies has produced a dependable and high-quality genomic resource. The value of this dataset is further enhanced by its clear organization and comprehensive documentation. The purpose of the dataset is explicitly stated, and the methods and materials are described in sufficient detail to ensure reproducibility. Additionally, the data are standardized, supported by clear visual representations, and made readily accessible, making this resource broadly applicable for diverse research purposes. 

 In addition to its intrinsic scientific value, the genome of *H*. *fagi* holds notable practical significance. While the preservation of natural ecosystems remains a central priority in conservation, this species is particularly sensitive to land-use abandonment and the subsequent encroachment of vegetation, which can alter or degrade its habitat. In this context, the availability of a high-quality genomic resource provides an important foundation for both fundamental and applied research. It enables a deeper understanding of the species’ biology, population dynamics, and adaptive potential, thereby supporting the development of more informed and effective conservation and management strategies.

Are sufficient details of methods and materials provided to allow replication by others?

Yes

Is the rationale for creating the dataset(s) clearly described?

Yes

Are the datasets clearly presented in a useable and accessible format?

Yes

Are the protocols appropriate and is the work technically sound?

Yes

Reviewer Expertise:

Evolutionary Biology, Ecology, IPM, Integrative Biology, Entomology, Genomics

I confirm that I have read this submission and believe that I have an appropriate level of expertise to confirm that it is of an acceptable scientific standard.

# Reviewer report: Reviewer response for version 1

**DOI:** 10.21956/wellcomeopenres.28477.r152251

**Published:** 2026-04-15

**Authors:** Vlad Eugen Dinca

**Affiliations:** 1 University of Oulu Centre of Excellence in Cell-Extracellular Matrix Research, Oulu, Northern Ostrobothnia, Finland

In this note, the authors have successfully generated a chromosome-level genome assembly of a male *Hipparchia fagi *(Lepidoptera: Nymphalidae). The mitochondrial genome has been assembled as well.

 Although I am not an expert in several of the methodological approaches used, as far as I can tell, the manuscript is technically sound and uses methodologies and pipelines that are fairly well-established through use by the Wellcome Sanger Institute.

 The genome appears to be of high quality and scaffolded into 29 chromosomal pseudomolecules (including the Z sex chromosome).

 A few additional comments are included below.

 You are mentioning that it closely resembles other *Hipparchia species*. It would be good to briefly mention whether it can be reliably distinguished from those based on external characters, genitalia and/or DNA barcodes.

 “from northern Spain (Cantabrian Mountains) across southern to western Kazakhstan and the southern Urals.” – In northern Spain, this species occurs outside the Cantabrian Mts. as well (e.g. the sequenced specimen originates from NE Spain). Perhaps this sentence could be slightly rephrased. A suggestion:

 “..... from northern Spain across southern Europe to the southern Urals and western Kazakhstan.”

 “It has one generation per year, with adults starting to fly in early June in southern Europe, diapause in summer and re-appear again in September”- Slight rephrasing suggested: “It has one generation per year, with adults starting to fly in early June in southern Europe, diapausing in summer and re-appearing again in September”

 Figure 1 – The upper image shows forewings (ventral) and hindwings (dorsal and upside down); the lower image shows the forewings (dorsal) and hindwings (dorsal and upside down). This should ideally be corrected to first show all wings dorsally and in the correct position, followed by all wings shown ventrally in the correct position.

Are sufficient details of methods and materials provided to allow replication by others?

Yes

Is the rationale for creating the dataset(s) clearly described?

Yes

Are the datasets clearly presented in a useable and accessible format?

Yes

Are the protocols appropriate and is the work technically sound?

Yes

Reviewer Expertise:

Lepidoptera speciation, phylogeography, conservation.

I confirm that I have read this submission and believe that I have an appropriate level of expertise to confirm that it is of an acceptable scientific standard.
